# The effect of human recombinant epidermal growth factor on neovascularization and pedicle division time in a rat interpolation flap model

**DOI:** 10.55730/1300-0144.5812

**Published:** 2024-01-05

**Authors:** Aziz PARSPANCI, Yavuz KEÇECİ, Peyker TEMİZ

**Affiliations:** 1 Department of Plastic, Reconstructive and Aesthetic Surgery, Bayburt State Hospital, Bayburt, Turkiye; 2 Department of Plastic, Reconstructive and Aesthetic Surgery, Manisa Celal Bayar University, Manisa, Turkiye; 3 Department of Pathology, Manisa Celal Bayar University, Manisa, Turkiye

**Keywords:** Interpolation flap, pedicle division, recombinant human epidermal growth factor

## Abstract

**Background/aim:**

In practice, waiting 2–3 weeks for interpolation flaps pedicle division result in certain morbidities and discomfort for patient. The division time of flap pedicle depends on neovascularization from the recipient bed and includes wound healing stages. We aimed to investigate the effect of recombinant human epidermal growth factor (rhEGF) on the flap viability during early pedicle division.

**Materials and methods:**

Thirty-six rats were allocated to two main groups as control and study. A cranial based flap measuring 5 × 5 cm was elevated from the back, including all layers of the skin. While the cranial half of the defect was primarily closed, the flap was inset into the distal half. In the study group, a single dose of 20 μg EGF was injected into the recipient site and wound edges before the flap inset. The control group received no treatment. Each main group was divided into three subgroups based on pedicle division time of 8, 11 and 14 days. After pedicle division, each flap was monitored and photographed for 7 days, and histopathological samples were collected. Viable and necrotic areas were compared, and flaps were examined histopathologically.

**Results:**

The necrosis area in the study group on the 11th day was significantly lower than that in the control group. The fibroblastic activity, granulation tissue and neovascularization on the 8th day, the granulation tissue level on the 11th day, and the neovascularization level on the 14th day were significantly higher in the study groups.

**Conclusion:**

Following the application of EGF, the necrosis area decreased within the study group. Histopathological assessments revealed a statistically significant increase in parameters related to granulation tissue and fibroblastic activity, notably neovascularization, across all subgroups within the study. It was concluded that the use of EGF positively affected the neovascularization, and flaps could be divided earlier.

## 1.Introduction

Interpolation flaps, widely used in plastic and reconstructive surgery, are preferred for large and deep defects resulting from trauma, congenital defects, tumor excisions, and burns, especially in cases where the surrounding tissue is inadequate for closure of these defects [1,2].

The interpolation flap procedure is a two-stage procedure, and the patient may experience morbidities such as pain, limitations of motion, decreases in joint range of motion, and dysfunction in the postoperative period until flap division [1,3]. If pedicle division can be performed earlier, it is possible to restore functions and shorten the exposure time to these morbidities. This is important in terms of saving time, enabling an early return to work, and enhancing patient comfort [1,3].

The division of pedicles in interpolation flaps results from neovascularization from the recipient bed. This process includes stages of wound healing [3,4]. In the literature, there are studies on accelerating neovascularization and dividing flaps earlier. These include the use of adipose cell-derived mesenchymal stem cells [1], fibroblast growth factor [5], hyperbaric oxygen therapy [3], ischemic gradient creation [6], and the use of vascular endothelial growth factor (VEGF) [7].

EGF plays an important role in regulating cell growth, proliferation, and differentiation, as well as accelerating wound healing [8]. Studies have shown that exogenously administered EGF positively affects wound healing by increasing granulation tissue growth, cell proliferation, and migration [9–11]. Additionally, EGF has been shown to accelerate neovascularization [11–14]. Therefore, we thought that this effect of EGF could potentially shorten flap division time.

The objective of this study was to investigate the effect of EGF application on neovascularization in the recipient bed, recipient wound edges, and pedicle division time in an interpolation flap model previously described by Izmirli et al. [1].

## 2. Materials and methods

The study was approved by Manisa Celal Bayar University Faculty of Medicine Animal Experiments Local Ethics Committee (MCBU HADYEK) (dated 03/06/2020 and numbered 17250; research file). In the study, male and female Wistar albino rats, aged 36 weeks and weighing from 150 to 250 g, were randomly selected without regard to gender. All rats underwent a one-week adaptation period to ensure they were free from any health issues. Throughout the study, the animals were kept under stable conditions at 22 °C, with humidity ranging from 30 to 70% and subjected to a 12/12-h light/dark cycle in the treatment center. Standard rat chow and tap water were provided ad libitum to all animals.

In the experimental model, the animals were divided into two main groups, the control (K) group (n = 18) and the study (L) group (n = 18), each comprising 18 animals, without regard to weight and gender. Furthermore, each of these main groups was divided into three subgroups.

**Control (K) group (n = 18):** not treated with rhEGF.

K1 group: not treated with rhEGF, pedicle divided on the 8th day (n = 6).
K2 group: not treated with rhEGF, pedicle divided on the 11th day (n = 6).
K3 group: not treated with rhEGF, pedicle divided on the 14th day (n = 6).

**Study (L) group (n = 18):** rhEGF was applied.

L1 group: rhEGF was applied, pedicle divided on the 8th day (n = 6).
L2 group: rhEGF was applied, pedicle divided on the 11th day (n = 6).
L3 group: rhEGF was applied, pedicle divided on the 14th day (n = 6).

Following intramuscular anesthesia with a combination of ketamine (75 mg/kg) and xylazine (10 mg/kg), the rats were positioned prone, and the hair was shaved from the cervical to the distal region along the midline of the back. A cranial-based flap measuring 5 × 5 cm, containing a central artery and vein, was drawn.

**Figure 1:**
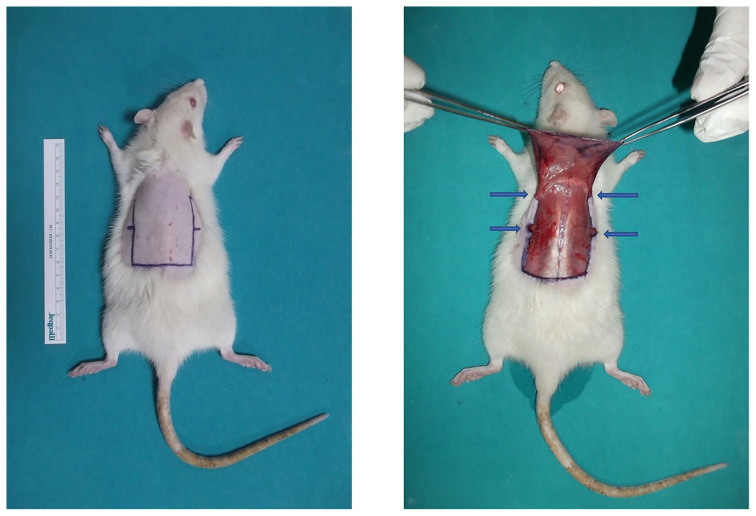
Flap design (a), flap elevation. Blue arrows: 1 cm incisions made at the midpoint of the defect at the base and to the lateral sides in the most proximal region (b).

Markings were made on the lateral lines corresponding to the middle of the flap. Consequently, the area to be repaired and the boundaries of the defect area were determined, as shown in Figure 1a. Beginning from the caudal end, the flap was dissected from the plane below the panniculus carnosus to cranial side, as shown in Figure 1b.

**Figure 2:**
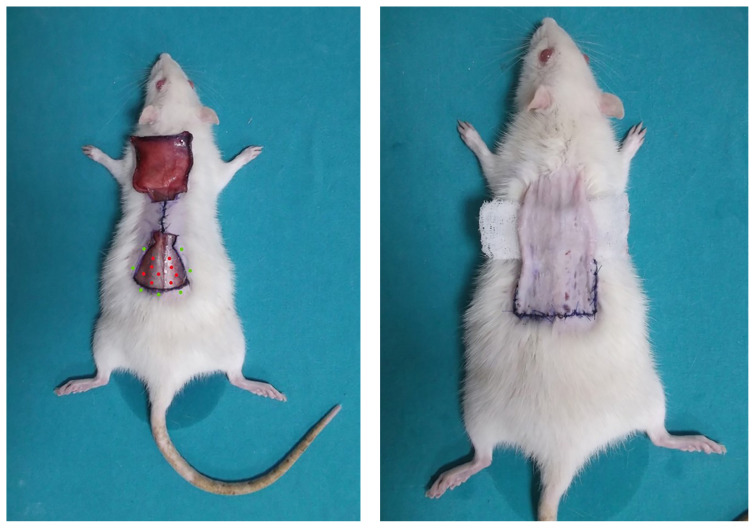
Closure of the proximal defect area. Red dots: recipient bed base, green dots: recipient bed wound edges (a), pedicle dressing (b).

After flap elevation, the proximal half of the defect was primarily repaired using 4/0 nonabsorbable suture material, as shown in Figure 2a. Before primary closure, two incisions were made on each side of the defect: one at the level of the flap base and another at its midpoint, as shown in Figure 1b. Thus, pedicle curling was substantially reduced.

All animals in the three subgroups (L1, L2, L3) of the study (L) group (n = 18) were administered a single injection of 20 μg of rhEGF immediately preceding the flap inset during the initial surgical procedure. The recombinant human epidermal growth factor (rhEGF) is marketed under the trade name ‘Heberprot-P’ and provided in vials containing 75 μg. It is produced by the Center for Genetic Engineering and Biotechnology (CIGB) in Havana, Cuba, and distributed by Praxis Havana/Cuba Pharmaceutical SA in Vitoria, Spain. It is derived from a genetically modified strain of Saccharomyces cerevisiae at the Center for Genetic Engineering and Biotechnology, and consists of a mixture of EGF1–51 and EGF1–52 forms [15].

Using an insulin injector, 15 μg were homogeneously injected into the recipient bed base, and 5 μg were homogeneously injected into the wound edges of the recipient bed, as shown in Figure 2a. Throughout the injection process, a visible bulge was formed on both the recipient bed base and the wound edges of the recipient bed.

After repairing the base area and applying rhEGF, the distal side of the flap was inset on the base defect using 4/0 nonabsorbable suture material. Antibiotic ointment-impregnated gauze was placed between the pedicle and primary repaired area at the base to prevent the pedicle from drying out, as shown in Figure 2b. Dressing was applied every other day.

**Figure 3:**
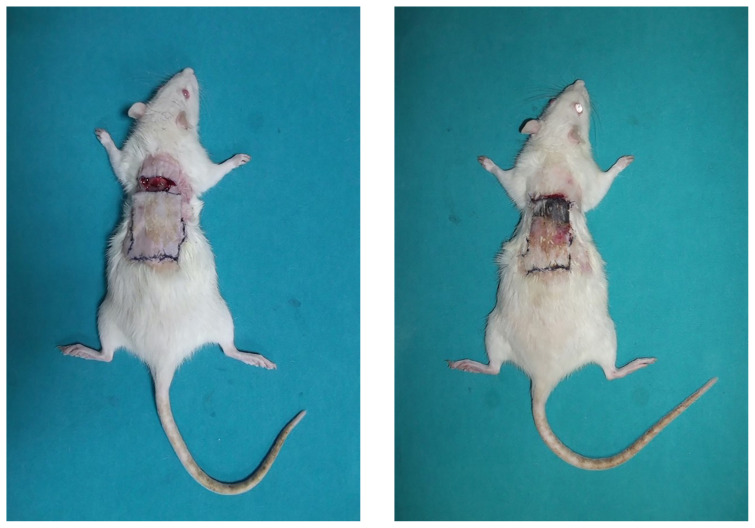
Pedicle division (a), 7th day after pedicle division (b).

After the follow-up periods, the pedicles were divided on the 8th day in the K1 and L1 groups, on the 11th day in the K2 and L2 groups, and on the 14th day in the K3 and L3 groups, as illustrated in Figure 3a. To prevent flap contraction after the division of the proximal pedicles, the flaps were fixed in the same places with 3–4 simple sutures.

### 2.1. Macroscopic evaluation

**Figure 4:**
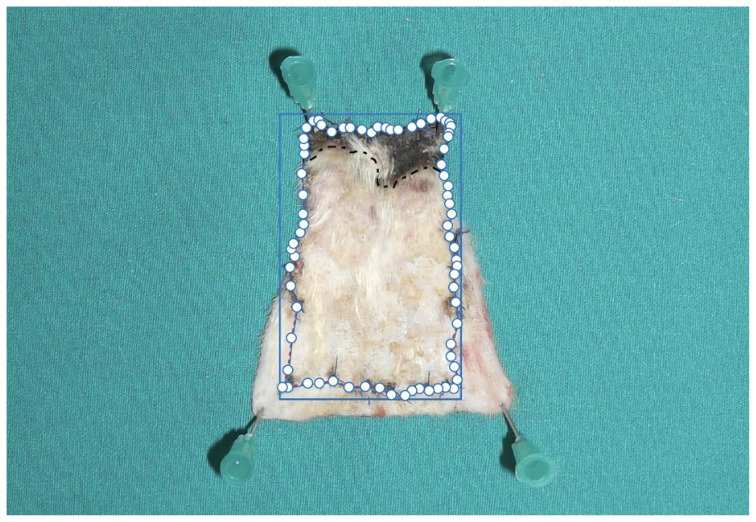
Measurement of living and necrotic areas.

Measurements were made one week after the pedicles of the flaps were divided, as shown in Figure 3b. Following the administration of high-dose anesthesia to all animals, samples including at least 1 cm of intact tissue from the suture line of the distal flap were obtained. These samples were affixed onto a green background using needle tips and then transported to the pathology laboratory for histopathological examination. All photographs were taken with a digital camera (Fujifilm FinePix AV210, 5.7–17.1 mm, Japan) from a distance of 50 cm under suitable light conditions. All photographs were imported to the Adobe Acrobat Reader DC 2021 program. The viable areas and necrosis areas of the flaps in the photograph were measured in pixels, and the necrotic flap area relative to the entire flap area was determined as a percentage (%) as shown in Figure 4. Graphic representations of all flaps were generated based on these measurements.

### 2.2. Microscopic evaluation

**Figure 5:**
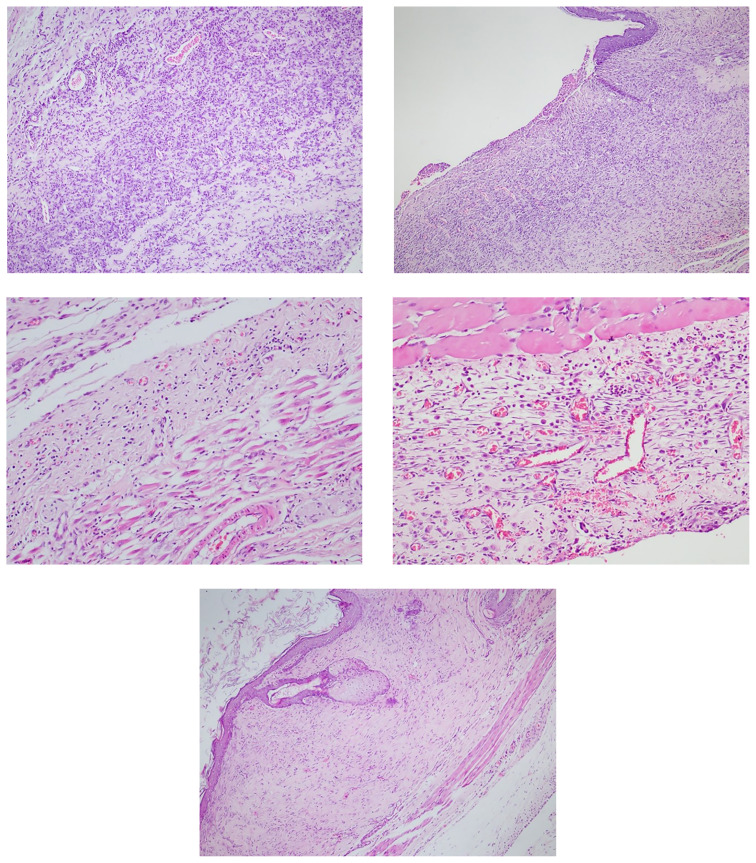
Neovascularization, inflammation, fibroblastic activity, granulation tissue. Grade 3 (HE, ×100) (a), reepithelialization. Grade 2 (HE, ×40) (b), neovascularization, inflammation, fibroblastic activity, granulation tissue. Grade 1 (HE, ×100) (c), neovascularization, inflammation, fibroblastic activity, granulation tissue. Grade 2 (HE, ×100) (d), full repair, granulation. Grade 4 (HE, ×40) (e).

The samples obtained from the back of each animal were kept in 10% formaldehyde for 24 h before being transported to the pathology laboratory. Subsequently, each sample was evaluated macroscopically and microscopically by a single pathologist. After recording the size and general appearance of the samples, the tissues were divided into three areas, with particular focus on the caudal section where the sutures were located, and 4–6 samples were taken from each area. After paraffin embedding, 5-μm sections were prepared and stained with hematoxylin-eosin. The sections were evaluated by the same pathologist using the BX53F light microscope with a blinded approach, wherein the pathologist was unaware of which group they belonged to. The caudal area, where the flap was attached to the normal skin, was scored. Reepithelialization, inflammation, fibroblastic activity, granulation tissue, and neovascularization findings in these samples were evaluated using a semiquantitative method. The findings were scored according to the values outlined in Table 1. Microscopic images corresponding to these scores are provided in Figures 5a–5e.

### 2.3. Statistical method

The obtained data were transferred to the computer environment and analyzed using the statistical package program SPSS version 15.0 (SPSS Inc., Chicago, IL, USA). Descriptive statistics were presented as numbers, percentages, and mean values. Subgroups comparisons within groups were conducted using the Freidman test, while the comparison between subgroups among groups was performed using the Mann-Whitney U test. The statistical significance level was determined to be p≤0.05.

## 3. Results

### 3.1. Macroscopic findings

In Table 2, the flap necrosis areas measured one week after pedicle division of the subjects in the groups are presented in pixels and percentages (%). The mean and standard deviation of each group are indicated in Table 2.In the control group, the mean postoperative necrosis area was 388.90 ± 83.90 mm^2^ on day 8, 269.8 ± 37.98 mm^2^ on day 11, and 179.61 ± 96.41 mm^2^ on day 14. The observed differences were statistically significant (p = 0.009). In the study group, the mean postoperative necrosis area was 337.80 ± 173.99 mm^2^ on day 8, 181.5 ± 51.25 mm^2^ on day 11, and 167.34 ± 86.18 mm^2^ on day 14. However, the differences were not statistically significant (p = 0.115), as detailed in Table 2.

**Figure 6:**
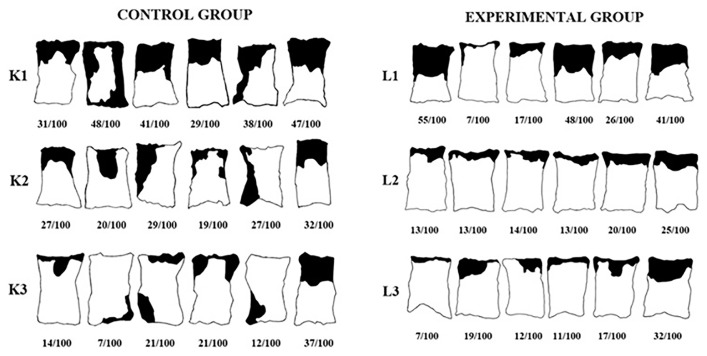
Graphic images of the subjects’ flap living areas and necrosis areas with percentage measurements.

**Figure 7:**
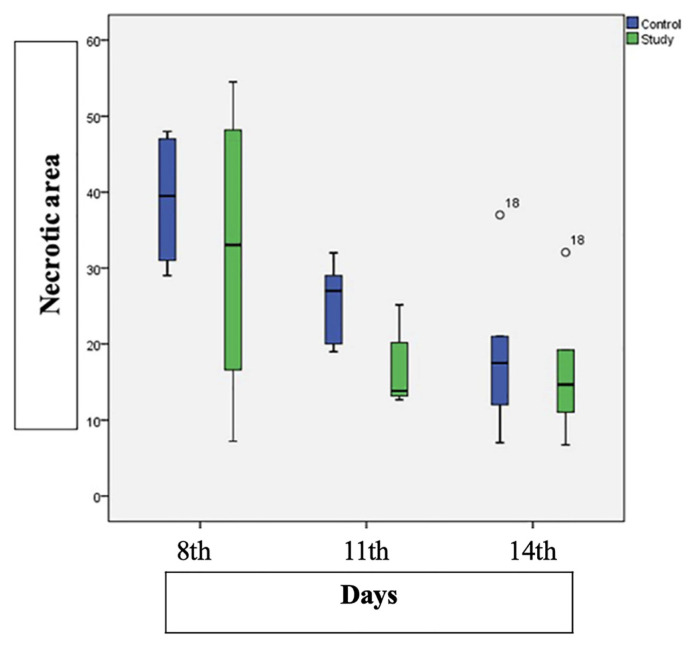
Percentage distribution of necrosis areas in rats from both the study and control groups, measured according to pedicle division days.

The statistical p values for flap necrosis areas in the subgroups of both the control and study groups, as determined by the Mann-Whitney U test, were 0.699 for the 8th day, 0.015 for the 11th day, and 0.589 for the 14th day.At the conclusion of the study, the necrosis and viable areas observed within the flaps of the subjects in each group were digitized on photographs, as shown in Figure 6. Notably, in the control group, a significant decrease in the percentage of necrosis area was observed with an increase in the postoperative evaluation duration (p < 0.05). Conversely, within the study group, there was no significant difference in the percentage of necrosis area across the examination days (p > 0.05). There was no significant difference in the mean necrosis area between the subgroups divided on the 8th and 14th days (p = 0.699, p = 0.589).The mean necrosis area on the 11th day in the control subgroup was significantly higher than that in the matching subgroup of the study group (p = 0.015). The percentage distribution of necrosis areas in rats from both the study and control groups, measured based on pedicle division days, is depicted in Figure 7.

### 3.2. Microscopic findings

The scoring results from histopathologic evaluation of samples obtained from the subjects in the groups are presented in Table 3. On postoperative day 8, there was no significant difference between the control and study groups in terms of reepithelialization and inflammation (p > 0.05 for each). However, the study group exhibited significantly higher levels of fibroblastic activity, granulation tissue, and neovascularization (p < 0.05 for each). On postoperative day 11, there was no significant difference between the control and study groups in terms of reepithelialization, inflammation, fibroblastic activity, and neovascularization levels (p > 0.05 for each); however, granulation tissue level was significantly higher in the study group (p = 0.023).

On postoperative day 14, there was no significant difference between the control and study groups in terms of reepithelialization, inflammation, fibroblastic activity, and granulation tissue (p > 0.05 for each); however, the level of neovascularization was significantly higher in the study group (p = 0.027).

### 3.3. Statistical analyses

In comparing flap necrosis areas, when the control group was evaluated based on the time of pedicle division (8th, 11th, and 14th days), it was observed that necrosis area decreased significantly as time progressed (p = 0.009). However, when the same comparison was made in the study group, although there was a decrease in the total necrosis area, it did not reach statistical significance (p = 0.115).When comparing the areas of flap necrosis between the study and control subgroups, in which pedicle division was performed on the same days, significant results were obtained on day 11 (p = 0.015), while the results obtained on days 8 and 14 were not statistically significant (p = 0.699, p = 0.589).Histopathologic data were compared between the study and control subgroups in which pedicle division was performed on the same day.

Fibroblastic activity, granulation tissue, and neovascularization levels (p < 0.05 for each) in the study group in which the pedicle was divided on day 8, granulation tissue level (p < 0.05) in the study group in which the pedicle was divided on day 11, and neovascularization (p < 0.05) in the study group in which the pedicle was divided on day 14 were significantly higher than the control subgroups in which pedicle division was performed on the corresponding days.

## 4. Discussion

Studies have shown that the duration of pedicle division in interpolation flaps depends on neovascularization from recipient bed [4,6]. In the literature, there are many studies on pedicle division and neovascularization. The studies aimed to understand the physiology of neovascularization, to shorten pedicle division time, and consequently to provide patient comfort by preventing comorbidities related to the surgical procedure [4,16,17].In general, the current conventional rationale of reconstructive surgeons favors waiting three weeks before flap division [6,18–22]. Many surgeons appear to find greater satisfaction in waiting for three weeks and obtaining more assured results compared to the temptation of pedicle division a few days earlier [18]. Despite this appearing to be a standardized time, the time of pedicle division actually varies based on factors such as the patient’s age, nutrition status, smoking habits, and additional morbidities (e.g. diabetes, prior radiotherapy). The optimum time of pedicle division is determined by the surgeon, considering all these factors. Therefore, there is no clear standardization regarding the time of pedicle division [6,18,23,24]. Most studies in the literature aim to shorten the pedicle division time and/or safely divide the pedicle at the optimal time.Gatti et al. emphasized that the evaluation of neovascularization and the timing of tube flap model division can enhance the use of fluorescent material. This method can aid in the follow-up of neovascularization and prove useful in determining the optimal time for pedicle division, thus shortening the pedicle division time [18]. Calloway et al. utilized laser-assisted indocyanine green angiography on patients undergoing forehead flap surgery, aiming for earlier pedicle division, and consequently, a reduction in total cost [17]. A similar study was performed by Rudy et al. and similar results were obtained [16]. These types of studies show the development of circulation. However, they have disadvantages such as requiring an additional procedure and cost, as well as the equipment not being widely available.Tsur et al. studied the timing of pedicle division in skin flaps among rats and pigs. They concluded that the development of new vascular channels was sufficient to maintain the overall survival of flaps after 6 to 7 days in rats. In the same study on pigs, it was demonstrated that neovascularization, sufficient for flap survival at the recipient site, originated from both the wound edges and the base of the recipient bed. However, vascular channels developing from the base of the recipient bed held significantly greater significance [4].Based on the results of this study, three-fourths of the rhEGF was applied to the base of recipient bed, while one-fourth was applied to the wound edges of the recipient bed in our study.The results of some studies in the literature indicate that pedicle division time may exceed normal durations in patients with comorbidities and wound healing issues. Therefore, particular attention should be given to pedicle division time in these patients [6,25]. Stark et al. investigated the effect of low perfusion in the wound edges on neovascularization development in a rat tube flap model [25]. Additionally, Park et al. emphasized the significance of ischemic gradient between the interpolation flap and recipient bed in determining flap viability [6].Upon reviewing the literature, it is seen that there are many clinical and experimental studies on EGF. In general, these studies focus on examining the effects of EGF on wound healing, granulation tissue stimulation, and epithelialization. rhEGF is currently used in wound healing in humans, particularly in diabetic wounds, and this positive effect on wound healing can be utilized in flap viability.There are not enough studies in the literature investigating the effect of EGF on flap viability. It is known that pedicle division time in interpolation flaps depends on the development of neovascularization from the recipient bed, and this process includes stages of wound healing. Thus, the 20 μg dose of EGF used by Hong et al. in the rat full-thickness wound healing model was used as a reference in our study [26]. Additionally, the study by Tuluy et al. utilized the same dose of rhEGF to investigate its effect on capsule contraction in rats [27].In our study, we compared the parameters of reepithelialization, inflammation, fibroblastic activity, granulation tissue formation and neovascularization between the control and study groups. Our aim was to evaluate the effect of the procedure on neovascularization and pedicle division time by applying 20 μg rhEGF to recipient bed and wound edges.In the literature, studies on experimental models of staged flap transfer procedures have aimed to induce vascularization through exogenous interventions and to divide pedicle earlier. Khouri et al. concluded that the application of angiogenic factors can increase flap survival by accelerating revascularization [5]. Richards et al. also concluded that the application of hyperbaric oxygen therapy in an animal with a tube flap model induced angiogenesis from the flap recipient bed and increased tolerance to ischemia in the flap after pedicle division [3]. Zhang et al. showed that VEGF treatment causes neovascularization, increased blood flow and pressure, and increased tissue viability in various chronic ischemia models [7]. In their study utilizing an interpolation flap model in rats, Izmirli et al. observed that the application of adipose tissue-derived mesenchymal stem cells applied to the recipient bed and wound edges significantly increased neovascularization, as evidenced by both histopathological and scintigraphic analyses [1].In our study utilizing the interpolation flap model in rats, we observed that the use of EGF significantly increased the viable flap area and decreased necrosis in subjects in which flap division was performed on the 11th day. When examining the results obtained from histopathological evaluation of the study, it was observed that fibroblastic activity, granulation tissue, and neovascularization levels were significantly higher in the group in which pedicle division was performed on the 8th day, granulation tissue level in the group in which pedicle division was performed on the 11th day and neovascularization levels in the group in which pedicle division was performed on the 14th day. While the higher flap survival rates were different among subgroups in which flap division was performed on day 8, the absence of neovascularization level difference between these subgroups suggests that EGF provides benefits in terms of flap perfusion. Based on our study findings, the use of EGF in interpolation flaps can accelerate neovascularization, thus ensuring that the pedicle can be safely divided earlier.

## 5. Conclusion

Nowadays, interpolation flaps, which have relatively remained in the background with the development of microsurgical techniques, are still needed in various parts of body, particularly in the face and upper extremities, and less commonly in the lower extremities. In interpolation flaps, which have a staged procedure, the complications arising during the process, until pedicle division, as well as increased cost resulting from the treatment’s extended duration, can be prevented by shortening the pedicle division time.Numerous studies in the literature aim to increase neovascularization in interpolation flaps, and thus shortening the pedicle division time. However, there is a lack of sufficient publications examining the effect of EGF use on flap viability and further studies are required. Our study contributes to this field by potentially complementing existing research. The histopathologic data in our study were semiquantitatively evaluated, but more quantitative data could be obtained with different staining techniques and imaging methods.In conclusion, the use of EGF to accelerate neovascularization in interpolation flaps may contribute to earlier pedicle division, thus reducing the morbidities, workforce loss, and comfortless time of patients (especially with cross flaps) during this period, which may lead to positive social and economic results.

## Figures and Tables

**Table 1:** Scoring table used in microscopic evaluation.

**Reepithelialization**	0	No closure	**Inflammation**	0	No
	1	<30% closure		1	Mild
	2	31–60% closure		2	Moderate
	3	61–99% closure		3	Severe
	4	Complete closure			
**Fibroblastic activity**	0	No	**Granulation tissue**	0	No
	1	Mild		1	Thin
	2	Moderate		2	Medium
	3	Severe		3	Thick
	4	Fibroblastic activity accompanied by abundant collagen production		4	Full repair
**Neovascularization** **(Per square millimeter)**	0	0–4			
	1	5–14			
	2	15–24			
	3	>24			

**Table 2:** Results of the measured necrosis areas for all subjects in pixels and percentages.

RAT	K1		K2		K3		L1		L2		L3	
	mm^2^	%	mm^2^	%	mm^2^	%	mm^2^	%	mm^2^	%	mm^2^	%
**1**	357.64/1138.41	31	278.61/1036.57	27	147.76/1087.52	14	481.41/883.24	55	173.61/1318.41	13	54.14/805.09	7
**2**	519.39/1080.93	48	228.06/1122.95	20	63.65/942.5	7	90.76/1259.82	7	118.14/888.62	13	215.28/1119.26	19
**3**	408.31/985.95	41	306.56/1051.81	29	184.78/890.60	21	208.36/1255.71	17	177.61/1233.07	14	143.56/1199.42	12
**4**	271.12/933.57	29	219.92/1149.04	19	225.1/1090.92	21	545.68/1132.00	48	138.44/1093.10	13	107.43/972.71	11
**5**	350.08/922.45	38	277.06/1020.94	27	115.93/931.94	12	281.63/1102.00	26	250.62/1242.03	20	183.60/1058.38	17
**6**	426.83/899.43	47	308.56/975.04	32	340.43/931.52	37	418.95/1033.00	41	230.57/916.29	25	300.02/935.64	32
**Mean flap** **necrosis area**	**388,90**		**269,80**		**179,61**		**337,80**		**181,50**		**167,34**	
**Standard** **deviation**	**83,90**		**37,98**		**96,41**		**173,99**		**51,25**		**86,18**	

**Table 3:** Scoring results after histopathologic evaluation of sampled subjects.

Control group						Experimental group					
Experiment no	Reepithelialization	Inflammation	Fibroblast	Granulation tissue	Neovascularization	Experiment no	Reepithelialization	Inflammation	Fibroblast	Granulation tissue	Neovascularization
**K1-1**	3	2	2	2	3	**L1-1**	4	1	4	4	3
**K1-2**	3	2	3	2	2	**L1-2**	3	3	3	3	3
**K1-3**	4	2	2	2	2	**L1-3**	3	1	4	4	3
**K1-4**	4	2	2	1	2	**L1-4**	4	3	3	3	3
**K1-5**	3	2	2	2	2	**L1-5**	3	3	3	3	3
**K1-6**	4	1	2	2	2	**L1-6**	4	1	4	4	3
**K2-1**	4	1	3	2	2	**L2-1**	4	2	2	2	2
**K2-2**	3	2	3	2	3	**L2-2**	3	2	3	3	3
**K2-3**	4	1	3	2	2	**L2-3**	4	2	4	4	2
**K2-4**	3	3	2	2	2	**L2-4**	4	2	3	3	3
**K2-5**	3	3	3	3	3	**L2-5**	4	1	4	4	2
**K2-6**	4	1	3	2	2	**L2-6**	3	2	3	3	3
**K3-1**	4	1	3	2	2	**L3-1**	3	2	3	3	3
**K3-2**	4	1	2	2	2	**L3-2**	4	1	4	4	2
**K3-3**	3	1	2	2	2	**L3-3**	4	1	4	4	3
**K3-4**	4	1	3	2	2	**L3-4**	4	2	3	2	3
**K3-5**	3	1	3	2	2	**L3-5**	3	2	2	2	3
**K3-6**	4	2	3	3	3	**L3-6**	4	2	3	2	3
